# Blood flow pattern in the ascending aorta after TAVI and conventional aortic valve replacement: Analysis using 4D-Flow MRI

**DOI:** 10.1186/1532-429X-17-S1-O68

**Published:** 2015-02-03

**Authors:** Ralf F Trauzeddel, Ulrike Loebe, Alex J Barker, Carmen Gelsinger, Christian Butter, Michael Markl, Jeanette Schulz-Menger, Florian von Knobelsdorff-Brenkenhoff

**Affiliations:** 1 Cardiology, Heart Center Brandenburg, Bernau, Germany; 2 Radiology, Northwestern University, Chicago, IL, USA; 3 Cardiology, ECRC, Charité University Medicine Berlin and HELIOS Clinics, Berlin, Germany

## Background

Transarterial aortic valve implantation (TAVI) is a valid alternative to treat aortic stenosis in patients with high surgical risk. The impact of TAVI on changes in blood flow dynamics in the ascending aorta has not been systematically analyzed. Using temporally resolved, three-dimensional, phase contrast MRI (4D-flow), we studied 3D hemodynamics in the ascending aorta (AAo) after TAVI and compared the results to patients with conventional aortic valve replacement (AVR) as well as healthy controls.

## Methods

We enrolled 17 subjects with TAVI (Edwards Sapien, mean age 77±7 years, 9 males), 12 with biological AVR (77±4 years, 8 males), and 9 healthy subjects (55±16 years, 8 males). All underwent 4D-flow MRI at 1.5T (spatial resolution 1.8 x 1.8 x 2.5mm^3^, temporal resolution 40.8ms). Analysis included quantification of the regional distribution of peak systolic wall shear stress (WSS) and peak systolic flow velocity in the AAo.

## Results

**Figure 1:**
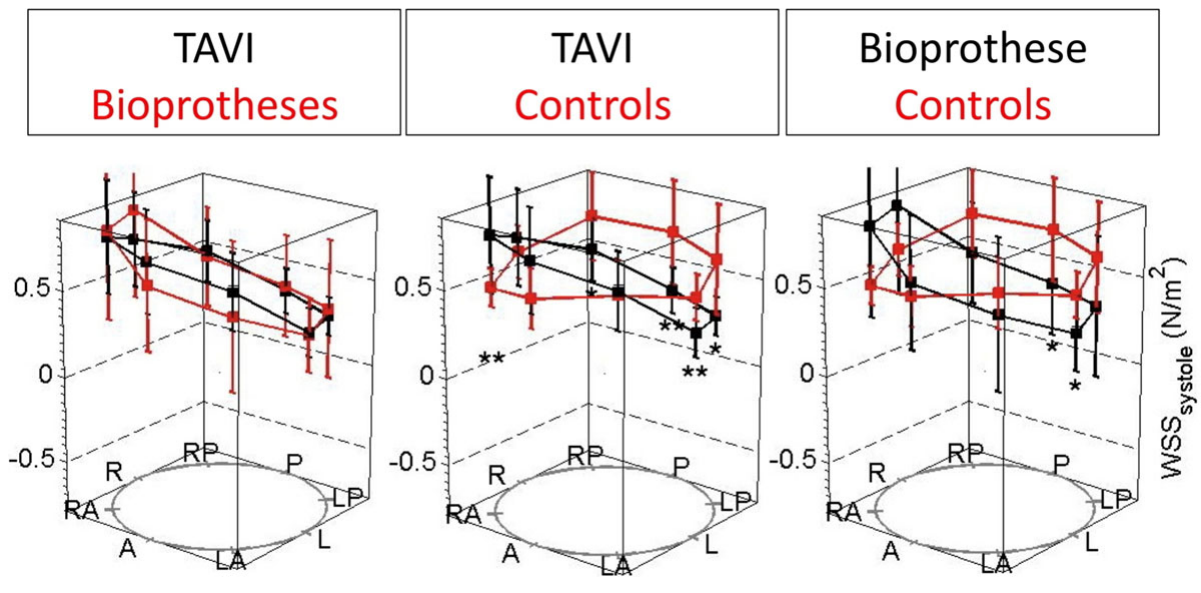
Distribution of wall shear stress in the ascending aorta (a=anterior, p=posterior, l=left, r=right).

**Figure 2:**
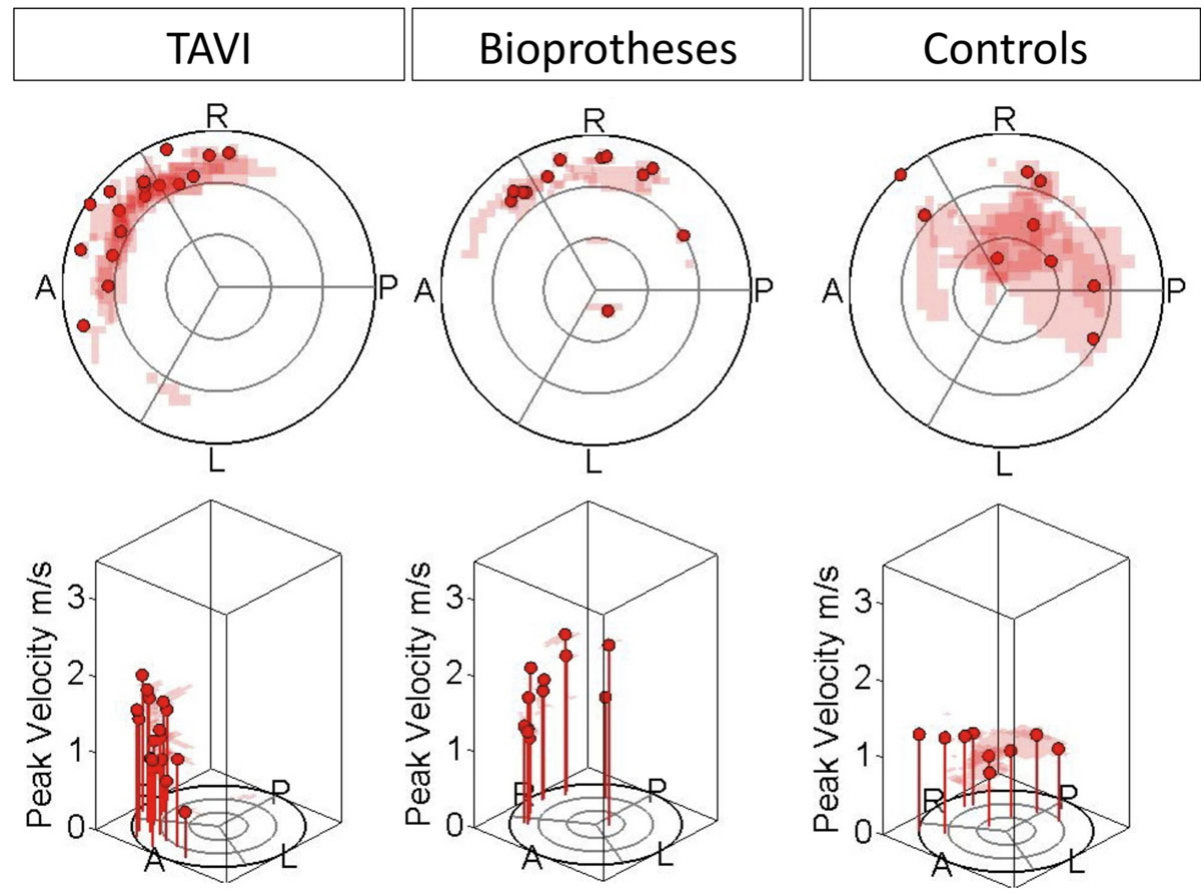
Distribution of peak blood flow velocity in the ascending aorta (a=anterior, p=posterior, l=left, r=right).

The mean aortic orifice area was 1.9±0.2cm^2^ (TAVI), 1.4±0.4cm^2^ (AVR) and 4.0 ±0.8cm^2^ (Controls) (p<0.001). Mean aortic diameter was 35±3mm, 39±4mm and 31±5mm (p=0.002). Patients with TAVI and AVR showed an asymmetric distribution of systolic WSS in the AAo compared to controls. WSS was significantly elevated anteriorly and right-anteriorly, and reduced posteriorly (Fig. 1). WSS distribution was similar in TAVI and AVR. Both TAVI and AVR exhibited the peak flow velocity along the right-anterior curvature, while this was more central in controls (Fig. 2). Peak velocities did not differ between TAVI and AVR (p=0.550).

## Conclusions

TAVI and AVR lead to similar alterations in systolic blood flow patterns in the ascending aorta compared to healthy controls.

## Funding

None.

